# Expression and Functional Analysis of *AMT1* Gene Responding to High Ammonia Stress in Razor Clam (*Sinonovacula constricta*)

**DOI:** 10.3390/ani13101638

**Published:** 2023-05-14

**Authors:** Chenxin Hu, Wenfang Dai, Xiaojie Zhu, Hanhan Yao, Zhihua Lin, Yinghui Dong, Liyuan Lv

**Affiliations:** 1 Key Laboratory of Aquatic Germplasm Resource of Zhejiang, College of Biological and Environmental Sciences, Zhejiang Wanli University, Ningbo 315100, China; 17658164586@163.com (C.H.); ahtlhuchenxin@163.com (X.Z.); yaohanhan1020@126.com (H.Y.); zhihua9988@126.com (Z.L.); 2 Ninghai Institute of Mariculture Breeding and Seed Industry, Zhejiang Wanli University, Ningbo 315604, China; daiwenfang6283@163.com

**Keywords:** AMT1, *Sinonovacula constricta*, ammonia excretion, RNA interference, gene expression, SNP validation

## Abstract

**Simple Summary:**

Ammonia (NH_3_/NH_4_^+^) is one of the most common contaminants in the aquaculture environment. Ammonium transporter 1 (AMT1) is a member of the Amt/Mep/Rh family that facilitates movement of ammonia across plasma membranes. However, the role of AMT1 in mollusks remains unclear. In this work, we found that the activation of AMT1 expression in razor clam (*Sinonovacula constricta*) can be significantly induced under high ammonia exposure, and AMT1 synergizes with Rhesus glycoprotein (Rh) to facilitate ammonia transport. The results will help to better understand the biological functions of AMT1 in razor clam ammonia excretion and improve studies of its molecular mechanism.

**Abstract:**

Ammonium transporter 1 (AMT1), a member of ammonia (NH_3_/NH_4_^+^) transport proteins, has been found to have ammonia transport activity in plants and microorganisms. However, the functional characteristics and molecular mechanisms of AMT1 in mollusks remain unclear. The razor clam (*Sinonovacula constricta*) is a suitable model species to explore the molecular mechanism of ammonia excretion because of the high concentration of ambient ammonia it is exposed to in the clam–fish–shrimp polyculture system. Here, the expression of *AMT1* in *S. constricta* (*Sc-AMT1*) in response to high ammonia (12.85 mmol/L NH_4_Cl) stress was identified by real-time quantitative PCR (qRT-PCR), Western blotting, RNA interference, and immunofluorescence analysis. Additionally, the association between the SNP_g.15211125A > T linked with *Sc-AMT1* and ammonia tolerance was validated by kompetitive allele-specific PCR (KASP). A significant upregulated expression of Sc-AMT1 was observed during ammonia exposure, and Sc-AMT1 was found to be localized in the flat cells of gill. Moreover, the interference with *Sc-AMT1* significantly upregulated the hemolymph ammonia levels, accompanied by the increased mRNA expression of Rhesus glycoprotein (Rh). Taken together, our findings imply that AMT1 may be a primary contributor to ammonia excretion in *S. constricta*, which is the basis of their ability to inhabit benthic water with high ammonia levels.

## 1. Introduction

Ammonia nitrogen is one of the most common harmful and toxic substances, which can lead to ion imbalance, oxidative stress, inflammatory response, hypoimmunity, growth slowdown, and high mortality in aquatic animals [1,2]. Ammonia primarily exists as two forms in the aqueous environment, ionic ammonia (NH_4_^+^) and nonionic ammonia (NH_3_), and the relative amounts of each are governed by the buffer reaction: NH_3_ + H_3_O^+^⇌NH_4_^+^ + H_2_O (pKa = 9.0–9.5) [3]. Accordingly, the vast majority of ammonia is present as NH_4_^+^ and only a very small amount is present as NH_3_ under physiological conditions where the pH is typically below the pKa [4]. Indeed, NH_3_ and NH_4_^+^ both have limited permeability across lipid bilayers [5]. As a consequence, excessive ammonia is typically excreted to the surrounding water by various transport proteins in aquatic invertebrates [6]. It has been proposed that ammonium transporters (AMTs) and Rhesus glycoproteins (Rhs) may be capable of transporting ammonia (NH_3_/NH_4_^+^) across cell membranes [3]. In addition, NH_4_^+^ has nearly identical biophysical characteristics to K^+^, which enables NH_4_^+^ to compete with K^+^ for essentially all K^+^ carrier proteins, such as Na^+^/K^+^-ATPase (NKA), Na^+^-K^+^-2Cl^−^ cotransporter (NKCC), and K^+^-channels [6]. Similarly, specific Na^+^/H^+^ exchanger (NHE) isoforms may function in Na^+^/NH_4_^+^ exchange mode and contribute to gill epithelial ammonia transport [3,6]. In this regard, aquatic organisms possess a complex network of ammonia transport, in which AMTs may serve as important channel proteins for ammonia transport and excretion.

AMTs were first discovered to transport NH_4_^+^ in plant roots [7]. The studies on AMTs have been mainly focused on its chemical structure, evolutionary relationship with Rh, and ammonia transport function in plants and microorganisms [8,9,10]. In contrast, there have been few studies on AMTs in invertebrates [11,12,13]. AMT is a homotrimer, and each subunit contains a hydrophobic pore primarily for NH_3_ conduction and an NH_4_^+^ binding site [9,14]. In scaly clam (*Tridacna squamosa*), AMT1 can be found at the epithelium of ctenidium [13], and there are indications of its active involvement in ammonia excretion [12,15]. Similarly, AMT1 was suggested to facilitate ammonia excretion in mosquito (*Aedes aegypti*) owing to a decrease in ammonia efflux in both larval anal papillae and a significant increase in ammonia content in hemolymph induced by double-stranded RNA (dsRNA)-mediated gene expression reductions in AMT1 [12]. Besides, ammonia excretion can also be mediated by Rh proteins, which function as gas channels for CO_2_/NH_3_ and ion transport channels for NH_4_^+^ in organisms [8,16]. In swimming crab (*Portunus trituberculatus*), the interference with *Rh* influenced the mRNA expression of ammonia-excretion-related genes including *NHE*, aquaporin (*AQP*), K^+^ channels, and vesicle-associated membrane protein (*VAMP*); increased the activity of NKA and V-type H^+^-ATPase (VHA); and significantly reduced the ammonia excretion rate, together with an increase in ammonia and glutamine (Gln) levels in hemolymph [17]. Thus, the results suggested that Rh protein may play an important role in ammonia excretion. Likewise, phylogenetic and bioinformatic analyses have demonstrated that Rh proteins and AMTs may have a paralogous relationship in early evolution as they coexist in many species of unicellular eukaryotic microbes and invertebrates [8].

In recent years, the clam–fish–shrimp polyculture system has developed into a new optimized model for aquaculture, but the residual food and fecal matter in this system can lead to constant accumulation of ammonia in the pond sediment [18]. For example, several investigations of seawater composition in intensive aquaculture ponds showed considerable concentrations of ammonia in a range between 100 and 500 µmol/L, but also up to 2.5 mmol/L in a sediment depth of 4–9 cm [19,20]. Therefore, the razor clam (*Sinonovacula constricta*), as a burrowing bivalve, is a suitable model species to explore the molecular mechanism of tolerance to ammonia in mollusks [20,21,22]. Genes such as glutamate dehydrogenase (*GDH*), glutamine synthetase (*GS*), glutamic oxaloacetic transaminase (*GOT*), glutathione S-transferase (*GST*), heat shock protein 90 (*HSP90*), and *Rh* showed distinct expressional patterns during continuous exposure to high ammonia in razor clams [22,23,24,25]. Furthermore, abundant expression of transport-, immune-response-, and apoptosis-related pathways was activated under ammonia stress. Recently, 56 associated single nucleotide polymorphisms (SNPs) (−log10*P* = 5), including the SNP (g.15211125A > T) adjacent to *AMT1* gene, were identified in the genome-wide association study (GWAS) of ammonia tolerance in *S. constricta*. Based on this, we inferred that *AMT1* gene might be a potential regulator of ammonia tolerance through its participation in ammonia excretion in razor clam. However, the molecular characteristics of AMT1 in *S. constricta* (Sc-AMT1) and its functions in response to ammonia stress were still unclear.

In this study, we firstly validated the genotypes of the significant SNP (g.15211125A > T) located at 2.6 kb upstream of *Sc-AMT1* by kompetitive allele-specific PCR (KASP), and then analyzed the molecular characteristics, temporospatial expression, and tissue localization of Sc-AMT1. Further, the changes in mRNA expression of *Rh* and hemolymph ammonia concentration were explored after the RNA interference (RNAi) of *Sc-AMT1*. The results will improve our understanding of Sc-AMT1 function on ammonia excretion and provide a potential marker gene for molecular-assisted breeding to improve ammonia tolerance in *S. constricta*.

## 2. Materials and Methods

### 2.1. Animal Cultivation

Healthy adult clams (wet weight 15.38 ± 1.84 g, shell length 59.36 ± 2.52 mm) were collected from Ningbo Marine and Fishery Science and Technology Innovation Base, Zhejiang Province, China, and then cultured in the Zhejiang Key Laboratory of Aquatic Germplasm Resources. Before the experiment, the clams were acclimated in tanks with 50 L aerated seawater (salinity 22 ± 1, pH 7.9 ± 0.1, ammonia-N 0.02 ± 0.01 mmol/L, nitrite-N 0.36 ± 0.06 μmol/L) at 20.0 ± 1.0 °C for one week. The seawater, composed of recrystallized sea salt, was filtered through an activated carbon cartridge before use and renewed (50%) every 12 h. The salinity, pH, ammonia-N, nitrite-N, and temperature were monitored by AZ8371 salinometer (Hengxing, Taiwan, China), HACH HQ30d pH meter (Hach Company, Loveland, CO, USA), HACH DR2008 portable spectrophotometer (Hach Company), and AQT-15 crystal thermometer (Voonline, Shenyang, China), respectively. The clams were fed twice per day at 8:00 and 18:00 with microalgae (Chlorella vulgaris), with a photoperiod of 12 h light/12 h dark.

### 2.2. Ammonia Challenge

Before the challenge treatment, six healthy clams were dissected to investigate the distribution of *Sc-AMT1* in different tissues including gill, hepatopancreas, intestine, siphon, mantle, foot, and adductor muscle (posterior and anterior adductor muscles). Based on the median lethal concentration (LC_50_-96 h) of ammonia-N (17.46 mmol/L) according to our previous toxicity tests [22], the clams were randomly divided into an experimental group (12.85 mmol/L NH_4_Cl) and control group (<0.02 mmol/L ammonia-N). Each group contained a total of 120 clams in three replicate tanks (40 clams in each tank). The ammonia reagent was prepared by dissolving NH_4_Cl and the concentration was maintained by replenishing NH_4_Cl every 12 h. During the experiment, the pH was maintained at 7.9 ± 0.1 for the buffer system of CO_2_/HCO_3_^−^ in the seawater. The treatment continued for 96 h. The gills of six individuals were randomly collected at 0, 3, 6, 12, 24, 48, 72, and 96 h after exposure to ammonia respectively, and immediately frozen in liquid nitrogen. The samples were then stored at −80 °C for total RNA and protein extraction. Finally, the gills of six surviving individuals from each group were collected at 96 h and fixed with 4% paraformaldehyde for paraffin section and immunohistochemistry testing.

### 2.3. Real-Time Quantitative PCR Analysis

Total RNA was extracted using a total RNA extractor (Sangon, Shanghai, China). RNA purity was measured using NanoDrop 2000 spectrophotometers (Thermo Fisher Scientific, Waltham, MA, USA). After the quality and quantity analysis, the cDNA from 1 μg of total RNA was synthesized using PrimeScript RT reagent Kit with gDNA Eraser (TaKaRa, Shiga, Japan). The primers were designed according to the coding sequences of *Sc-AMT1* (GenBank accession no. OQ244848), *Sc-Rh* (GenBank accession no. OQ244849), and ribosomal protein S9 (*RS9*) (GenBank accession no. OQ244850) by Primer Premier 5.0 software and synthesized by Sangon Biotech (Sangon, Shanghai, China) (Table 1). The specificity of primers was determined by the formation of single PCR products and melt curve analysis. The real-time quantitative PCR (qRT-PCR) reaction was performed in a total volume of 20 µL mixture containing 0.8 µL of cDNA, 1 µL of 10 µM forward primer, 1 µL of 10 µM reverse primer, 7.2 µL of PCR-grade water, and 10 µL of 2× ChamQ Universal SYBR qPCR Master Mix (Vazyme, Nanjing, China) with a Light Cycler 480 instrument (Roche, Basel, Switzerland). The amplification program for qRT-PCR was as follows: 95 °C for 10 min, followed by 40 cycles of 95 °C for 10 s and 60 °C for 1 min, and 72 °C for 7 min of extension. All samples were measured in technical triplicate. The relative mRNA expression levels of *Sc-AMT1* and *Sc-Rh* were normalized to *Sc-RS9*, which has a more stable expression than others in *S. constricta* [26], and subsequently calculated according to the standard 2^−∆∆CT^ method [27].

### 2.4. Temporal Expression of Sc-AMT1 by Western Blotting Analysis

The gill samples at different time points were added with an appropriate amount of Cell Lysis Buffer for Western and IP (Beyotime, Shanghai, China) and then centrifuged at 10,000× *g* for 10 min at 4 °C. The supernatant was collected to measure the protein concentration using the bicinchoninic acid (BCA) method with the Pierce™ BCA protein assay kit (Thermo Fisher Scientific), and an equal amount of protein (50 μg) was electrophoresed by sodium dodecyl sulfate polyacrylamide gel electrophoresis (SDS-PAGE). Subsequently, the target protein in gel was transferred to polyvinylidene fluoride (PVDF) membrane and then blocked for 1 h in blocking solution (5% skimmed milk powder) at room temperature, followed by primary antibody (anti-Sc-AMT1 antibody, produced privately by HuaBio, Hangzhou, China, 1:500) incubation (overnight, 4 °C). Next, the primary antibody was bound by secondary antibody (anti-rabbit labeled with biotin HRP (1:2000)) for 1.5 h. Finally, the enhanced chemiluminescence (ECL) substrate mixture was incubated with the PVDF membrane. The proteins were observed and photographed by ChemiDoc™ Touch Imaging System (Bio-Rad, Hercules, CA, USA). In addition, glyceraldehyde 3-phosphate dehydrogenase (GAPDH) was used as an internal loading control for data normalization [27]. The gray values of the bands were analyzed with Image J software (NIH, Bethesda, MD, USA). Western blots were performed for all individual samples per experimental treatment; however, only one representative blot film is shown in the figures (Supplementary Figure S1).

### 2.5. Sequence Analysis of Sc-AMT1

The open reading frame (ORF) of *Sc-AMT1* was predicted by NCBI ORF Finder (https://ncbiinsights.ncbi.nlm.nih.gov/tag/orffinder/, accessed on 5 April 2022). The amino acid sequence, protein molecular mass, and isoelectric point (pI) were predicted using Proteomics Server of Expert Protein Analysis System (Expasy) (http://expasy.org/, accessed on 5 April 2022). The protein phosphorylation sites were predicted by NetPhos (https://services.healthtech.dtu.dk/service.php?NetPhos-3.1, accessed on 5 April 2022). The homology search was performed with BLAST program (https://blast.ncbi.nlm.nih.gov/Blast.cgi, accessed on 5 April 2022). The PFAM domains and transmembrane regions were determined by SMART (http://smart.embl-heidelberg.de/, accessed on 6 April 2022). The secondary structure of the Sc-AMT1 protein was analyzed using the GOR IV Method (https://npsa-prabi.ibcp.fr/cgi-bin/npsa_automat.pl?page=/NPSA/npsa_gor4.html, accessed on 6 April 2022). The 3D protein structure model of Sc-AMT1 was generated by SWISS-MODEL Workspace (https://swissmodel.expasy.org/interactive, accessed on 6 April 2022). Multiple protein sequence alignments were performed with ClustalW by the DNAMAN software (Lynnon Biosoft, San Ramon, CA, USA). The mature peptides of Sc-AMT1 were used to construct phylogenetic trees with the neighbor-joining method using MEGA 7.0 (Arizona State University, Tempe, AZ, USA). Aligned sequences were bootstrapped with 1000 trials by Seqboot.

### 2.6. Paraffin Section and Immunofluorescence Staining

The gill samples fixed in 4% paraformaldehyde were dehydrated in gradient ethanol and transparentized using 1:1 xylene:anhydrous ethanol transparent reagent to prepare 4 µm thick paraffin tissue sections. After baking, the tissue sections were deparaffinized with xylene, rehydrated with gradient ethanol, washed with phosphate buffered saline (PBS), and retrieved with citrate antigen retrieval solution. Sections were then immersed in 5% bovine serum albumin (BSA, Sangon) for 1 h and incubated with the primary antibody (anti-Sc-AMT1, HuaBio, 1:400) overnight at 4 °C. The sections were labeled with secondary antibody Alexa Fluor 488 donkey anti-rabbit immunoglobulin G (HuaBio, 1:150) for 1 h. Finally, the nuclei were stained with 4′,6-diamidino-2-phenylindole (DAPI, Beyotime). The gill tissues were observed and photographed by the fluorescence microscope Eclipse 80i (Nikon, Tokyo, Japan).

### 2.7. RNA Interference Assay

The dsRNA sequences for *Sc-AMT1* and negative control dsRNA (NC, as a non-specific dsRNA control) were prepared using T7 RiboMAX Express RNAi Kit (Promega, WI, USA), with primers designed by SnapDragon-dsRNA Design tool (https://www.flyrnai.org/cgi-bin/RNAi_find_primers.pl, accessed on 18 April 2022) (Table 1). The synthesized dsRNA was subsequently purified using Monarch RNA Cleanup Kit (NEB, Waltham, MA, USA) and diluted to 0.22 μg/μL with diethylpyrocarbonate-treated water (DEPC-W). For in vivo RNAi, each clam was injected via the adductor muscle using a 50 μL syringe with 18.45 µL dsRNA-*AMT1* or 18.45 µL NC (negative control) or 18.45 µL DEPC-W (blank control) after a pre-experiment with three different dsRNAs for *Sc-AMT1* (Supplementary Figure S2). A total of 240 individuals (wet weight 13.38 ± 1.47 g, shell length 54.94 ± 3.62 mm) for RNA interference assay were acclimated; refer to ‘Animal Cultivation’ above. Clams were randomly classified into a control group (CG; normal seawater) and an ammonia stress group (AG; 12.85 mmol/L NH_4_Cl). dsRNA-*AMT1*, NC, and DEPC-W were injected into 40 individuals in each group respectively. No death was observed throughout the experiment. The pH values were between 7.8 and 8.0 during the challenge. Gills and hemolymph were collected at 0, 6, 12, 24, and 48 h post-injection. RNA was extracted from the gill samples and used to detect the efficiency of RNAi within 48 h after injection using qRT-PCR. The hemolymph samples were immediately centrifuged at 700× *g* for 10 min at 4 °C and the supernatant was collected as the serum sample and frozen at −80 °C for ammonia concentration analysis.

### 2.8. Determination of Hemolymph Ammonia Concentration

The ammonia concentration in hemolymph was determined according to the instructions of the blood ammonia assay kit (No. A086-1-1, Nanjing Jiancheng Bioengineering Institute, Nanjing, China). Hemocyanin was precipitated with protein precipitant to block enzyme activity, prevent the production of free ammonia, and remove most of the substances that interfered with color. Ammonia was prepared in protein-free filtrate by the Berthelot reaction. In this non-enzymatic ammonia assay protocol, ammonia is used to form indophenol, a highly colored product easily quantifiable by colorimetry (λ = 630 nm) using a plate reader. Then, the ammonia concentration in hemolymph was calculated by comparison to the standard solution.

### 2.9. SNP Validation

A total of 854 healthy adult clams (wet weight 13.79 ± 1.28 g, shell length 56.87 ± 4.61 mm) were exposed to 12.85 mmol/L NH_4_Cl and the treatment lasted for 180 h. The dead clams were recorded and collected every 2 h. The validation panel comprised 300 razor clams including the 150 individuals that died initially (18–96 h) under ammonia exposure (ammonia sensitive group, SG) and the 150 individuals that survived at the end (180 h) of the experiment (ammonia tolerant group, TG). The foot tissues were taken separately and stored at −80 °C for genomic DNA isolation and genotyping. Total DNA was extracted using the marine animal tissue genomic DNA extraction kit (TianGen, Beijing, China), and DNA purity was measured using NanoDrop 2000 spectrophotometers (Thermo Fisher Scientific).

The genotype data were generated using KASP assay. KASP primers of SNP_g.15211125A > T (Table 1) were designed by PolyMarker (http://www.polymarker.info/, accessed on 15 September 2022) and synthesized by Sangon Biotech. KASP assay was carried out in 384-well formats and set up as 10 μL PCR reaction volumes consisting of 5 μL of FLu-Arms 2× PCR Mix, 0.2 μL of KASP primer assay mix (VideGene, Guangzhou, China), 1 μL of 20 ng/μL genomic DNA, and 3.5 µL of deionized water. PCR was conducted with an ABI QuantStudio 12K Flex Real-Time PCR System (Life Technology, New York, NY, USA) following the manufacturers’ recommendations. Non-template controls (NTCs) were included in each run. KASP amplification was performed using the following thermal cycling profile: step 1: 94 °C, 15 min; step 2: 94 °C, 20 s, 61 °C, 1 min, 10 cycles in total; step 3: 94 °C, 20 s, 55 °C, 1 min, 26 cycles in total. The fluorescence measurement was taken for KASP genotyping after PCR amplification. The genotypes of samples were recorded using Intellics software (LGC Douglas Scientific, Alexandria, VA, USA).

### 2.10. Statistical Analysis

All statistical analyses were performed with SPSS 26.0 statistical package (IBM, Armonk, NY, USA). All experimental data were presented as mean ± standard deviation (SD). The difference in the genotype frequency of SNP locus between SG and TG was examined using the chi-square (χ^2^) test. The Kolmogorov–Smirnov test was used to assess the normality and homogeneity of variance of all of the data. The data with normal distribution were further statically analyzed using one-way ANOVA with the least significance difference (LSD) test. For all comparisons, a *p*-value < 0.05 was considered statistically significant.

## 3. Results

### 3.1. SNP Validation

A single marker corresponding to *Sc-AMT1* gene was validated with the respective individual phenotypic data. The favorable allele (A:A) for SNP_g.15211125A > T revealed 144 tolerant and 107 sensitive individuals, while the alternate unfavorable allele (T:T) observed 6 tolerant and 26 sensitive individuals. The heterozygote (A:T) showed perfect association with the phenotypic data for ammonia sensitivity. The comparison between the TG and SG showed that the genotype frequencies of SNP_g.15211125A > T were significantly different (*p* < 0.001) (Table 2).

### 3.2. Deduced Amino Acid Sequence and Phylogenetic Analysis of Sc-AMT1

**Figure 1:**
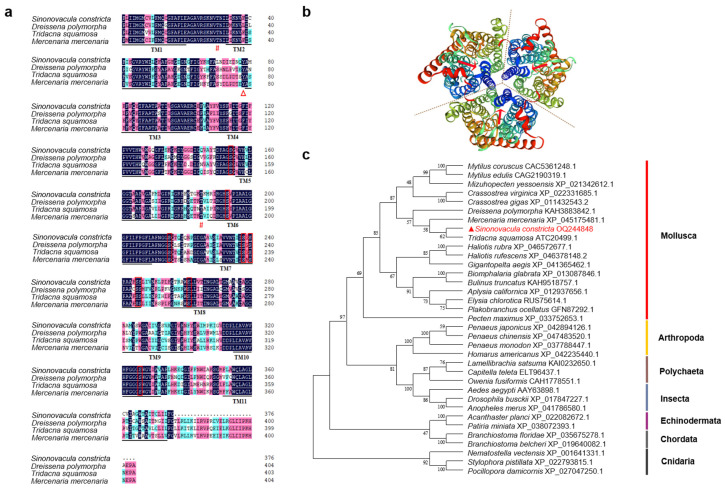
Multiple alignments of ammonium transporter 1 (AMT1) amino acid sequences, 3D space structure model, and phylogenetic tree of AMT1 amino acid sequences. (**a**) Alignments generated by ClustalW of AMT1 from *Sinonovacula constricta* with that of *Tridacna squamosa*, *Mercenaria mercenaria*, and *Dreissena polymorpha*. The 11 predicted transmembrane regions (TM1–TM11) are underlined and in bold. Vertical boxes, triangles, and hash marks represent the conserved amino acid residues including the serine, tyrosine, and threonine sites, respectively. (**b**) Threefold symmetry axis is indicated by dotted lines, and the red arrows indicate the channel position of each monomer. Transmembrane helices are progressively colored blue to red, indicating succession from N to C terminus. (**c**) Phylogenetic tree of AMT1 from *S. constricta* and other species is generated based on the neighbor-joining method. The percentage concordance based on 1000 bootstrap iterations is shown on the nodes.

The cDNA sequence of *Sc-AMT1* was submitted to GenBank (accession no. OQ244848). The complete sequence of cDNA was 4122 bp, containing a 5′ untranslated region (UTR) of 165 bp and an open reading frame (ORF) of 1653 bp, followed by a 3′ UTR of 234 bp. The ORF of *Sc-AMT1* encoded a protein of 550 amino acids residues with a calculated molecular mass of 59.67 kDa and theoretical pI of 5.54. The Sc-AMT1 contains 11 transmembrane regions (TMs) and a conserved ammonium transporter domain (24–429 amino acids) (Figure 1a). There is no N-terminal signal peptide in Sc-AMT1. A total of 31 phosphorylation sites were discovered including 17 serine, 10 threonine, and 4 tyrosine sites. Analysis on the amino acid sequence with GOR IV revealed that alpha helix was the major secondary structure in Sc-AMT1, accounting for 38.00%, followed by random coil (36.91%), extended strand (19.09%), and beta sheet (6.00%). The tertiary structure showed that Sc-AMT1 forms stable trimers on the membrane and has a conduction pore in each monomeric subunit (Figure 1b). The phylogenetic tree was divided into four main clades, of which mollusca and cnidaria were separately clustered into one clade, while arthropoda, polychaeta, and insecta were clustered together into one clade, and echinodermata was clustered together with chordata (Figure 1c). Notably, Sc-AMT1 was firstly clustered together with AMT1 from *T. squamosa*, followed by *Dreissena polymorpha* and *Mercenaria mercenaria*.

### 3.3. Expression Analysis and Tissue Localization of Sc-AMT1

**Figure 2:**
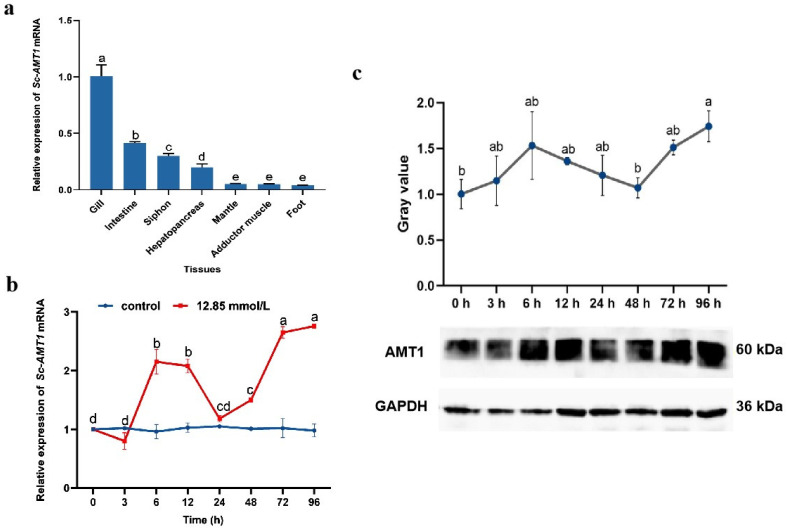
Quantitative expression of ammonium transporter 1 in *Sinonovacula constricta* (Sc-AMT1). (**a**) Tissue expression profiles of *Sc-AMT1*. (**b**) Temporal expression of *Sc-AMT1* mRNA in the gills after exposure to 12.85 mmol/L NH_4_Cl for 96 h. The control group was cultured in normal seawater. (**c**) Western blotting analysis of glyceraldehyde 3-phosphate dehydrogenase (GAPDH) and Sc-AMT1 in the gills under exposure to 12.85 mmol/L NH_4_Cl. The relative expression of Sc-AMT1 protein was obtained by the gray value of AMT1/GAPDH. Values are expressed as mean ± SD (n = 6). Different lowercase letters show significant difference among groups using one-way ANOVA (*p* < 0.05).

The mRNA expression level of *Sc-AMT1* was observed in all seven tested tissues (Figure 2a). Taking the foot as a control, the highest level of *Sc-AMT1* mRNA expression was observed in the gills (24-fold increase), followed by the intestine, with significantly lower expression in the mantle, foot, and adductor muscle (*p* < 0.05).The mRNA expression profile of *Sc-AMT1* in gill tissues was evaluated when exposed to 12.85 mmol/L NH_4_Cl. Specifically, the mRNA expression of *Sc-AMT1* in gill under ammonia stress significantly increased at 6 h and 12 h (*p* < 0.01), then decreased to the initial level (0 h), and eventually upregulated gradually from 48 to 96 h with a peak at 96 h (1.76-fold increase compared with 0 h) (Figure 2b). Similar to the mRNA expression pattern, the protein expression of *Sc-AMT1* in gill increased at 0–6 h, reduced at 6–48 h, and then upregulated at 48–96 h (Figure 2c).

**Figure 3:**
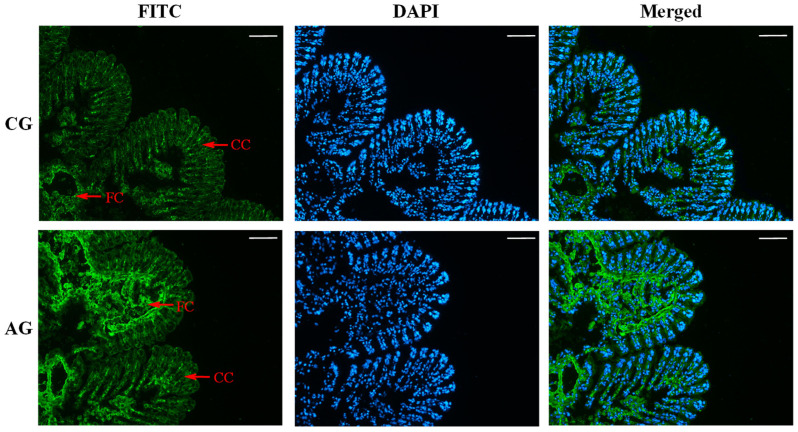
Immunofluorescence of ammonium transporter 1 (AMT1) in the gills of *Sinonovacula constricta* in the control group (CG; normal seawater) and ammonia stress group (AG; 12.85 mmol/L NH_4_Cl). The AMT1 was stained in green and nuclei stained with DAPI display blue fluorescence. FC, flat cell. CC, columnar cell. Scale bars are 50 µm.

The DAPI staining showed that there were many columnar cells in the gill filaments, and about half of the area near the central lumen consisted of flat cells (Figure 3). According to the green fluorescence signal, Sc-AMT1 in the flat cells in AG showed a stronger positive signal than that in CG. However, there was no significant difference in the columnar cells between AG and CG (Figure 3).

### 3.4. Effects of Sc-AMT1 RNAi on the Rh Expression and Hemolymph Ammonia Concentration

**Figure 4:**
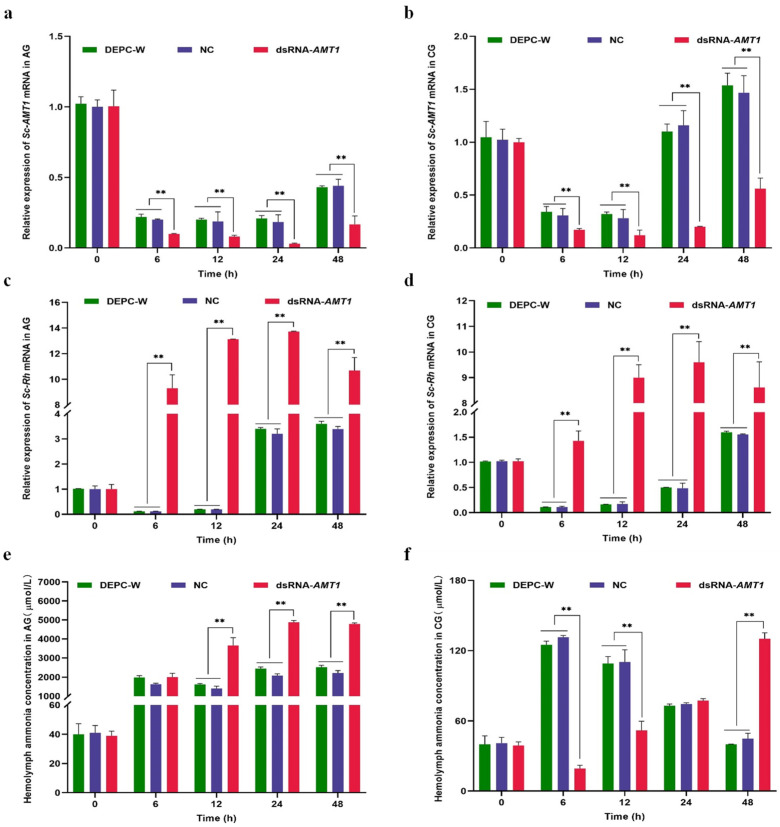
Relative expression of ammonium transporter 1 in *Sinonovacula constricta* (*Sc-AMT1*) in the gills after RNA interference (RNAi) and its effect on Rhesus glycoprotein (*Rh*) mRNA expression and hemolymph ammonia concentration. Time-course expression of *Sc-AMT1* genes in the ammonia stress group (AG; 12.85 mmol/L NH_4_Cl) (**a**) and control group (CG; normal seawater) (**b**) after RNA interference. Relative mRNA expression of *Rh* gene in AG (**c**) and CG (**d**) and hemolymph ammonia concentration in AG (**e**) and CG (**f**) of *S. constricta*. Vertical bars represent mean ± SD (n = 6). ** denotes an extremely significant difference between double-stranded RNA of *AMT1* (dsRNA-*AMT1*) and the control including the negative control dsRNA (NC, as a non-specific dsRNA control) and diethylpyrocarbonate-treated water (DEPC-W) based on one-way ANOVA (*p* < 0.01).

RNAi was used to study the effect of *Sc-AMT1* silencing on ammonia excretion in healthy clams. The interference efficiencies were significant at 6, 12, 24, and 48 h in each group (AG and CG), with 50%, 56%, 84%, and 62% in AG and 44%, 57%, 83%, and 62% in CG, respectively (Figure 4a,b). A significant decrease in the *Sc-AMT1* mRNA expression was detected at 6–24 h and then it increased markedly at 48 h in AG (Figure 4a). Similarly, *Sc-AMT1* expression was downregulated significantly at 6–12 h in CG (*p* < 0.01) and then increased gradually until 48 h (Figure 4b).

The *Sc-AMT1* silencing affected the mRNA expression of *Sc-Rh* in razor clams. Here, the expression level of *Sc-Rh* was significantly higher than that in the DEPC-W and NC groups during 6–48 h in AG after *Sc-AMT1* RNAi (*p* < 0.01), with a maximum at 24 h (3.29-fold increase compared with NC and DEPC-W), and the pattern of change of *Sc-Rh* was contrary to that of *Sc-AMT1* (Figure 4c). In addition, the *Sc-Rh* mRNA expression in CG showed a significant increase after the injection of dsRNA-*AMT1* (*p* < 0.01), and reached a peak at 24 h (18.80-fold increase compared with NC and DEPC-W) (Figure 4d).

The hemolymph ammonia concentrations in the *AMT1*-silenced group were significantly higher than those in both the NC and DEPC-W groups at 12–48 h in AG, with the peak value of 4878 μmol/L at 24 h (2.34-fold increase compared with NC and DEPC-W) (Figure 4e). The hemolymph ammonia concentration in the *Sc-AMT1* RNAi group began to decrease significantly compared with that in the NC and DEPC-W groups during 6–12 h in CG (*p* < 0.01), remained at the same level at 24 h, and then gradually increased until 48 h, with a minimum of 19.33 μmol/L at 6 h and a maximum of 130.15 μmol/L at 48 h (Figure 4f). In addition, there was no significant difference between the DEPC-W and NC groups in the above indices during the experiment (Figure 4a–f).

## 4. Discussion

Ammonia is one of the most common contaminants in the aquaculture environment, and its accumulation can cause a variety of hazards to organisms [1]. Thus, it is necessary to convert ammonia to non-toxic endogenous substances or rapidly discharge it to avoid its excessive accumulation in the body [28]. Remarkably, as benthic animals are often faced with higher ammonia concentrations compared with pelagic animals, they have evolved several mechanisms to adapt to high ambient ammonia [19]. In fact, excellent ammonia tolerance has been found in many marine bivalves, especially *S. constricta*, which lives under 30–40 cm of mud in ponds with an extreme high ammonia concentration [22,29]. A majority of aquatic invertebrates are ammonotelic and excrete ammonia primarily in the form of NH_3_ or NH_4_^+^, rather than relying on ammonia metabolism [19,30]. Increasing evidence has revealed that AMTs play a critical role in ammonia transport and are also involved in the ammonia absorption in plants, bacteria, and fungi [31,32]. However, there is a lack of information on the function of AMTs’ response to ammonia stress in invertebrates, especially in benthic bivalves. Therefore, we investigated gene expression profiles of *Sc-AMT1* in response to acute ammonia exposure in razor clam.

### 4.1. Association between the SNP and Ammonia Tolerance

In this study, the SNP located at 2.6 kb upstream of *AMT1* gene (Chr3: 15176152-15208520) identified from GWAS was verified by KASP. More recently, studies have shown that intergenic regions contain functionally important elements such as miRNAs with their own promoters, although these regions were once considered unimportant [33]. Despite the low overall level of intergenic sequence conservation, structural RNA elements within the intergenics are involved in the regulation of proximal gene expression [34]. For example, the strong association between opioid addiction and the SNP rs3754729 localized in the intergenic region between two circadian rhythm-related genes *PER2* and *HES6* was reported in European ancestry from the USA, supporting the role of SNP in long non-coding RNA (lncRNA) adjacent to circadian genes in the susceptibility to opioid addiction [35]. Here, a significant association between the SNP (g.15211125A > T) and ammonia tolerance in razor clam was found. We speculate that the conserved region around the SNP (g.15211125A > T) represents a regulatory region controlling constitutive expression of *Sc-AMT1*, and that the T allele leads to decreased expression of *Sc-AMT1*, thus affecting the response to ammonia stress in clams.

### 4.2. Sequence Characteristics and Phylogenetic Relationship of Sc-AMT1

AMT1 is a stable trimer on the cytoplasmic membrane and has a narrow channel within each monomer [14]. NH_4_^+^ deprotonation occurs after binding to a site on the periplasmic end of AMT1 and then passive diffusion of NH_3_ occurs through the strongly hydrophobic channels [9]. Our study identified that Sc-AMT1 contained a putative NH_4_^+^ binding site located at the base of the periplasmic vestibule and a conduction pore in each monomeric subunit. However, in the absence of a reliable in vitro assay, the conduction mechanism remains poorly understood. Furthermore, BLAST and ClustalW analysis indicated that the deduced amino acid sequence of Sc-AMT1 showed significant similarity to the AMT sequences from other species, excluding vertebrates, which was consistent with previous studies suggesting that the presence of AMT in animals may be, so far, limited to invertebrates [8,36].

### 4.3. Spatio–Temporal Expression Pattern of Sc-AMT1

Here, *Sc-AMT1* showed the highest expression in the gills, which is in accordance with the previous study in *T. squamosa*, suggesting *Sc-AMT1* plays an important role in the gills [13]. In aquatic animals, excessive ammonia is typically excreted into the surrounding water via the gills, and this process is mainly facilitated by AMTs and Rh proteins [6,36]. It has also been confirmed that the interference with *AMT1* in *A. aegypti* can increase the hemolymph ammonia concentration and significantly decrease the ammonia excretion rate [12]. In this study, *Sc-AMT1* seemed to be sensitive to ammonia exposure, because its transcript expression in the gills was significantly upregulated after ammonia accumulation, which was similarly demonstrated by Western blotting at the protein level. This result can be explained by the notion that the body may raise the efficiency of ammonia transport from the extracellular fluids through regulating the expression of *AMT1*, thereby reducing the toxicity of ammonia. The gill serves as an important organ that can perform ammonia excretion, as well as exchange of gas and ions between the internal fluids and external environment, and maintain the acid–base balance in aquatic organisms [37]. A variety of proteins participating in substances transport have been detected in gill, including AMT, Rh, NKA, NKCC, NHE, VHA, and carbonic anhydrase (CA) [38,39,40]. However, it is still unclear how AMT interacts with other proteins to regulate the excretion of ammonia in mollusks. We found that AMTs are broadly present in bacteria, algae, and invertebrates, and can excrete NH_3_ and NH_4_^+^ into the external medium [8,41]. In this study, the expression level of Sc-AMT1 fluctuated, but generally increased during ammonia exposure.

### 4.4. Tissue Localization of Sc-AMT1 in Gill

With the exception of mammals and cartilaginous fish, a majority of nitrogenous waste from aquatic organisms such as amphibians, teleosts, and aquatic invertebrates is excreted directly as ammonia [30,38]. NH_3_ is readily converted to NH_4_^+^ in the aqueous environment and excreted from gill via the ion transport system [42]. Histochemistry revealed that the gill filaments consist mainly of a single layer of various types of epithelial cells (subdivided into columnar and flat cells according to their positions) and endothelial cells surrounding a central lumen and resting on a basement membrane [43]. It is worth noting that flat cells are mainly responsible for exchange of gases and ions between hemolymph and surrounding water, while columnar cells participate in substances’ transport as the main cell type in cilia, which has an essential role in capturing and transporting particles during feeding [44]. However, the molecular mechanism of passage through these cells is still controversial [45]. Ammonia excretion in gill requires many transport proteins, and the members from the AMT/methylammonium permease (MEP)/Rh family are involved in the ammonia transport in most organisms [46]. Studies on mosquitoes and *T. squamosa* have found that AMT1 was generally localized in the apical membrane of epithelial cells in anal papillae and gill filaments, respectively [13,15]. Similarly, Sc-AMT1 was found to be located in the flat cells of gill in this study. Meanwhile, the protein abundance of Sc-AMT1 in the flat cells was significantly enhanced under ammonia stress. These findings inspired us to speculate that Sc-AMT1 may be involved in ammonia excretion in the gills of razor clam.

### 4.5. Effects of Sc-AMT1 RNAi on Sc-Rh Expression and Hemolymph Ammonia Concentration

AMT and Rh proteins are responsible for the selective movement of NH_3_ or NH_4_^+^ across biological membranes, which is a necessary process for all organisms [47]. In contrast to vertebrates that only possess Rh proteins, many invertebrates are unique and express both AMT and Rh proteins [19,48]. Among insect larvae, the presence of both AMT and Rh proteins has been identified in fruit fly (*Drosophila melanogaster*), malaria mosquito (*Anopheles gambiae*), and *A. aegypti* [11,15,49,50]. The interference with *AMT* or *Rh* resulted in an ammonia transport reduction in the anal papillae of *A. aegypti* larvae [15]. Thus, AMT and Rh proteins both play a central role in the ammonia excretion.

It has been shown that the ammonia concentration in hemolymph increased significantly when *AMT1* or *Rh* was interfered by dsRNA injecting or soaking [12,17]. A study on *A. gambiae* has suggested that AMT1 facilitated the uptake of ammonia from the hemolymph into the epithelial syncytium as NH_4_^+^ or NH_3_ [11]. Here, the hemolymph ammonia concentration in the clams injected with NC or DEPC-W under 12.85 mmol/L ammonia stress showed similar values to that determined in our previous study (nearly 2000 μmol/L) [22]. However, there was a onefold increase in the hemolymph ammonia concentration after RNAi of *Sc-AMT1*, supporting the importance of Sc-AMT1 in ammonia transport. In addition, it has been found that the mRNA expression of *Sc-Rh* increased significantly in the gills during ammonia stress [22], suggesting that Sc-Rh proteins were involved in ammonia excretion in the gills of *S. constricta*. In this study, the expression of *Sc-Rh* increased significantly after Sc-AMT1 RNAi (*p* < 0.05), indicating that Sc-AMT1 may serve as an ammonia channel, thereby synergizing with Sc-Rh to facilitate ammonia excretion, and there may be a compensatory interaction between Sc-AMT1 and Sc-Rh. Similar findings have been reported in zebrafish (*Danio rerio*) and *P. trituberculatus*, revealing that high ammonia exposure resulted in significant changes in the mRNA expressions of factors related to ammonia excretion (*NKA*, *VHA*, *NHE*, and *AQP1a1*) in *Rh*-suppressed organisms [17,51]. In combination, ammonia excretion is regulated by a variety of proteins including AMTs.

Interestingly, *Sc-AMT1* expression in the control group is higher than in the ammonia treatment group at 24 h and 48 h (Figure 4a,b). In general, the ammonia transporter might also serve as an overflow valve, transporting ammonia back into the hemolymph, when clams are exposed to high external ammonia concentrations. In the northern house mosquito (*Culex pipiens*) with a direct hemolymph volume expansion (i.e., injection of PBS), *AQP* mRNA levels were altered, suggesting that the injection of fluid may affect the ion and water balance in the gills [52]. Therefore, the downregulation of *Sc-AMT1* mRNA was likely in part a response to minimizing ammonia inflows, which reflected physiological changes in the gills after the injection of fluid.

## 5. Conclusions

In summary, this study validated the association between SNP_g.15211125A > T (located at 2.6 kb upstream of *Sc-AMT1* gene) and ammonia tolerance. Further, we identified the molecular characteristics of *Sc-AMT1* and analyzed its mRNA expression profiles in different tissues. A significant upregulated expression of Sc-AMT1 was observed during 12.85 mmol/L ammonia exposure (*p* < 0.05), and Sc-AMT1 was found to be localized in the flat cells of gill with a significantly increase in protein abundance. In addition, *Sc-AMT1* silencing increased the hemolymph ammonia concentration and upregulated the expression levels of *Sc-Rh*, indicating that Sc-AMT1 plays an important role in the ammonia excretion network. However, we only explored the gene responses to acute ammonia in *S. constricta*, while future comparative studies will include multi-dimensional analysis including more treatment methods and detection indices.

## Figures and Tables

**Table 1:** Primers used in this study.

Primers	Primer Sequence (5′-3′)	Tm (°C)	Product Size (bp)	Genbank Accession
qRT-PCR				
*AMT1*-F	GTGGTTCGTGGGGTGTGA	59.5	246	OQ244848
*AMT1*-R	GGTAGGCGGGCTCGTTAT	60.0		
*Rh*-F	TGCGGACCACATCCAGAA	59.4	106	OQ244849
*Rh*-R	GGCGACGGACCCCATAAC	61.2		
*RS9*-F	TGAAGTCTGGCGTGTCAAGT	60.2	117	OQ244850
*RS9*-R	CGTCTCAAAAGGGCATTACC	60.4		
RNA Interference				
dsRNA-*AMT1*-F	GCAGCAAGAAUGUCACAAATT	\	\	OQ244848
dsRNA-*AMT1*-R	UUUGUGACAUUCUUGCUGCTT	\	\	
NC-F	UUCUCCGAACGUGUCACGUTT	\	\	\
NC-R	ACGUGACACGUUCGGAGAATT	\	\	\
SNP Validation				
*AMT1*-SNP-F1	GAAGGTGACCAAGTTCATGCTTCATTAGAAATAAAATGCCGCAGAA	58.7	83	\
*AMT1*-SNP-F2	GAAGGTCGGAGTCAACGGATTTCATTAGAAATAAAATGCCGCAGAT	58.2		
*AMT1*-SNP-R	AATAGGATGTAACGCCATTCCTTACCTTG	60.3		

**Table 2:** Comparison of genotype frequencies of the SNP_g.15211125A > T between the TG (n = 150) and SG (n = 150) clam groups classified by ammonia tolerance.

Locus	Genotype	Number/Genotype Frequency (%)		Allele	Allele Frequency (%)		χ^2^/*p*-Value	Variation Type
		TG	SG		TG	SG		
g.15211125A > T	AA	144/96	107/71.33	A	96	77	34.954/0.000 **	Transversion
	AT	0/0	17/11.34	T	4	23		
	TT	6/4	26/17.33					

Significant difference between the ammonia-tolerant group (TG) and ammonia-sensitive group (SG) was indicated with two asterisks (*p* < 0.01).

## Data Availability

The authors declare that we have submitted the sequence information to NCBI with the numbers OQ244848, OQ244849, and OQ244850. We have released the above data.

## Supplementary Materials

The following supporting information can be downloaded at https://www.mdpi.com/article/10.3390/ani13101638/s1. Figure S1: Original Western blots used for the analysis and representative blot composition; Figure S2: Efficiency of RNA interference (RNAi) of ammonium transporter 1 in *Sinonovacula constricta* (*Sc-AMT1*) in normal seawater at 24 h post-injection.
