# Supplementary material for: Expression and Functional Analysis of AMT1 Gene Responding to High Ammonia Stress in Razor Clam (Sinonovacula constricta)
Source: Animals (Basel). 2023 May 14;13(10):1638. doi: 10.3390/ani13101638 (PMC10215384; doi:10.3390/ani13101638)
Supplement: Supplementary file 1 [file animals-13-01638-s001.zip › Figure S1.pdf]

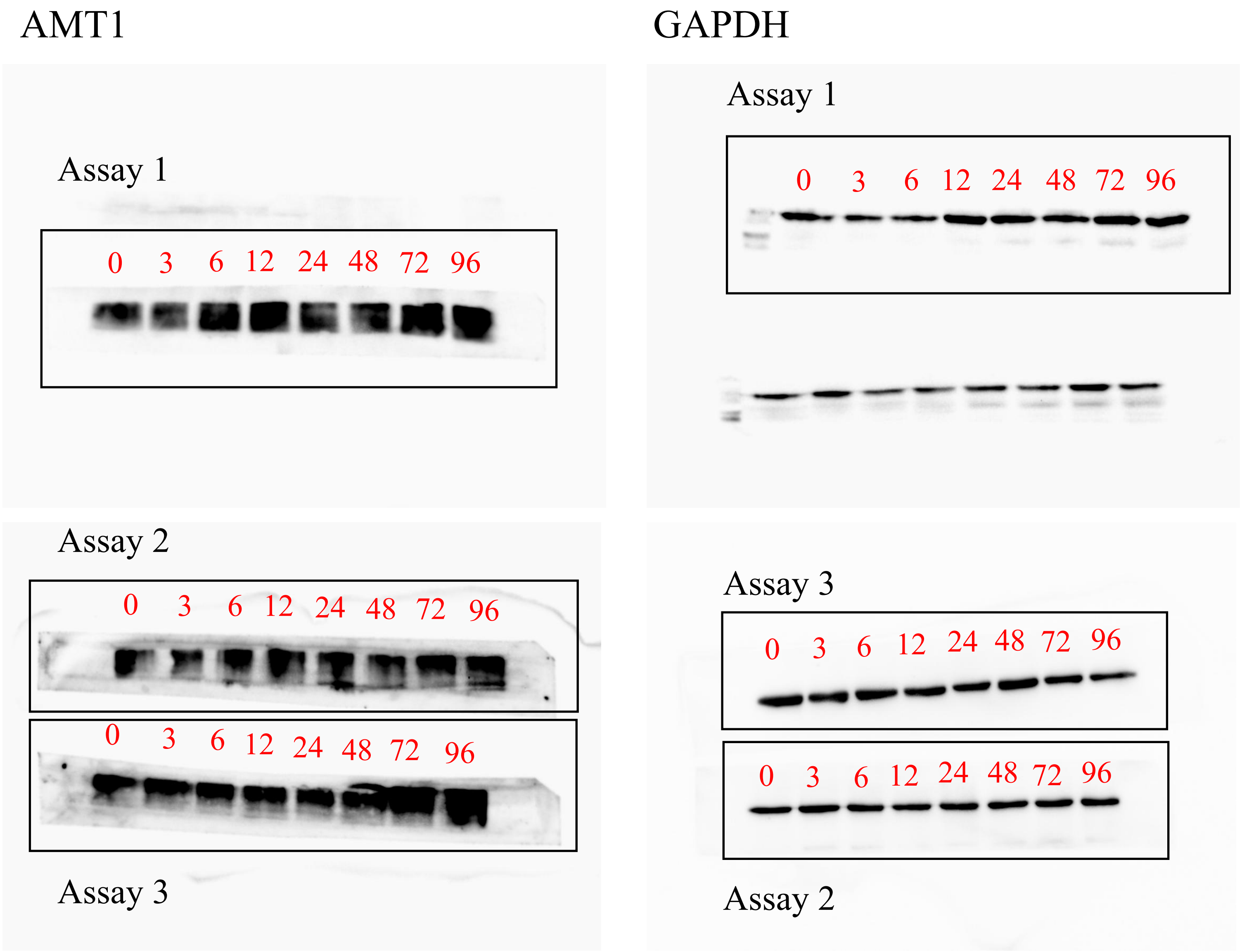


**Table S1. The densitometry readings of each band.**

| Time (h) | Assay 1 | | Assay 2 | | Assay 3 | |
|---|---|---|---|---|---|---|
| | AMT1 | GAPDH | AMT1 | GAPDH | AMT1 | GAPDH |
| 0 | 774484 | 365883 | 682701 | 285934 | 734585 | 426614 |
| 3 | 603319 | 204364 | 581922 | 321195 | 861219 | 360522 |
| 6 | 964398 | 238381 | 710253 | 243348 | 843151 | 332224 |
| 12 | 1068784 | 370167 | 716068 | 250480 | 898731 | 329621 |
| 24 | 785928 | 357599 | 869352 | 285848 | 691869 | 304434 |
| 48 | 846010 | 341064 | 618146 | 311045 | 895710 | 407922 |
| 72 | 1340437 | 417023 | 782755 | 267694 | 1043618 | 321884 |
| 96 | 1302773 | 387759 | 922861 | 269956 | 1112444 | 276348 |

**Supplementary Figure S1. Original Western blots used for the analysis and representative blot composition （n = 3）.**
