# Supplementary material for: Expression and Functional Analysis of AMT1 Gene Responding to High Ammonia Stress in Razor Clam (Sinonovacula constricta)
Source: Animals (Basel). 2023 May 14;13(10):1638. doi: 10.3390/ani13101638 (PMC10215384; doi:10.3390/ani13101638)
Supplement: Supplementary file 1 [file animals-13-01638-s001.zip › The explanation for Figure S1.pdf]

The explanation for figure S1

The original, uncropped and unadjusted images have been uploaded as supporting information in supplementary figure S1. The blots for each independent biological replicate used in the analysis have been provided in figure S1 (n =3). Additionally, Western blots were performed for all individual samples per experimental treatment and the gels of target protein AMT1 and GAPDH were incubated and visualized separately.
