# Supplementary figures and images for: Expression and Functional Analysis of AMT1 Gene Responding to High Ammonia Stress in Razor Clam (Sinonovacula constricta)
Source: Animals (Basel). 2023 May 14;13(10):1638. doi: 10.3390/ani13101638 (PMC10215384; doi:10.3390/ani13101638)

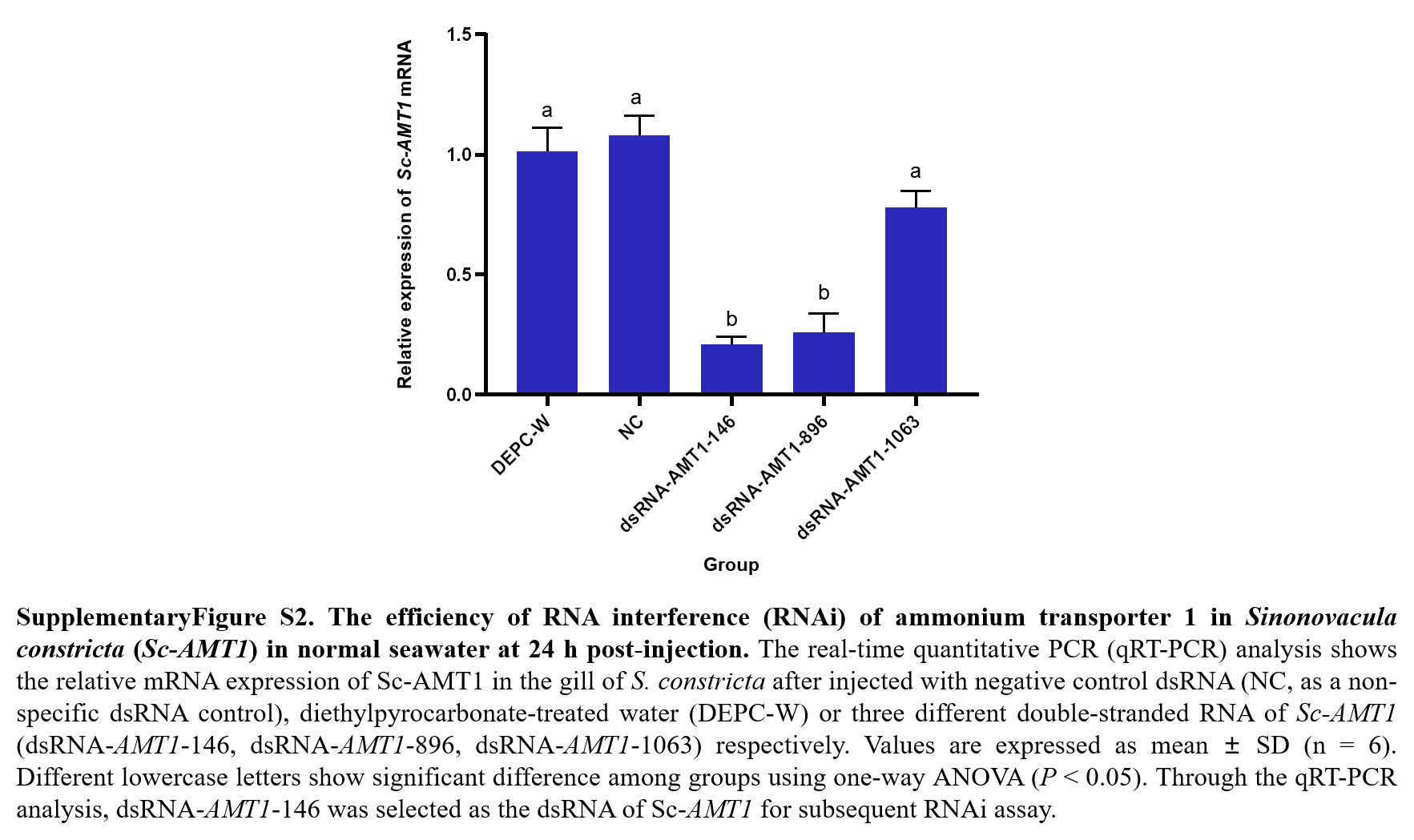

Supplement: Supplementary file 1 [file animals-13-01638-s001.zip › Figure S2.tif]
